# Characterization of MOSkin detector for *in vivo* skin dose measurement during megavoltage radiotherapy

**DOI:** 10.1120/jacmp.v15i5.4869

**Published:** 2014-09-08

**Authors:** Wei Loong Jong, Jeannie Hsiu Ding Wong, Ngie Min Ung, Kwan Hoong Ng, Gwo Fuang Ho, Dean L. Cutajar, Anatoly B. Rosenfeld

**Affiliations:** ^1^ Clinical Oncology Unit Faculty of Medicine, University of Malaya Kuala Lumpur Malaysia; ^2^ Department of Biomedical Imaging and University of Malaya Research Imaging Centre (UMRIC) Faculty of Medicine, University of Malaya Kuala Lumpur Malaysia; ^3^ Centre for Medical Radiation Physics (CMRP) University of Wollongong NSW Australia

**Keywords:** MOSFET, *in vivo* dosimetry, surface dose, skin dose, characterization, quality assurance (QA)

## Abstract

*In vivo* dosimetry is important during radiotherapy to ensure the accuracy of the dose delivered to the treatment volume. A dosimeter should be characterized based on its application before it is used for *in vivo* dosimetry. In this study, we characterize a new MOSFET‐based detector, the MO*Skin detector*, on surface for *in vivo* skin dosimetry. The advantages of the MO*Skin* detector are its water equivalent depth of measurement of 0.07 mm, small physical size with submicron dosimetric volume, and the ability to provide real‐time readout. A MO*Skin* detector was calibrated and the reproducibility, linearity, and response over a large dose range to different threshold voltages were determined. Surface dose on solid water phantom was measured using MO*Skin* detector and compared with Markus ionization chamber and GAFCHROMIC EBT2 film measurements. Dependence in the response of the MO*Skin* detector on the surface of solid water phantom was also tested for different (i) source to surface distances (SSDs); (ii) field sizes; (iii) surface dose; (iv) radiation incident angles; and (v) wedges. The MO*Skin* detector showed excellent reproducibility and linearity for dose range of 50 cGy to 300 cGy. The MO*Skin* detector showed reliable response to different SSDs, field sizes, surface, radiation incident angles, and wedges. The MO*Skin* detector is suitable for *in vivo* skin dosimetry.

PACS number: 87.55.Qr

## I. INTRODUCTION

Quality assurance (QA) in radiotherapy is very important in order to ensure the correct functioning of all components in radiotherapy, from treatment planning to the delivery of the treatment.^(1)^ Nowadays, advanced radiotherapy techniques, such as intensity‐modulated radiotherapy (IMRT), require patient‐specific QA to be performed to ensure the accuracy of radiation delivery during radiotherapy. However, these QA and verification procedures may not be sufficient to ensure the accuracy of the entire radiotherapy treatment.

A number of incidents have been reported recently.^2^, ^3^, ^4^ Human errors and systematic errors contributed to these incidents. Therefore, towards that end, *in vivo* dosimetry can detect major errors during the delivery of radiotherapy. It also can access clinical relevant differences between planned and delivered dose, record the dose received by the patient, and fulfill legal requirements.^(1)^

In radiotherapy, *in vivo* dosimetry means the measurement of the radiation dose received by a patient during treatment.^(1)^ Ideally, a dosimeter should be positioned at the point of interest inside a patient's body. However, in many cases it is not possible to place a dosimeter inside a real patient's body. Hence, the placement of a dosimeter on the surface of the patient's body becomes an alternative.

An ideal *in vivo* dosimeter should possess the following characteristics: (i) tissue equivalent; (ii) small in physical size and has small sensitive volume; (iii) features (e.g., temperature, energy) which are consistent and characterizable; (iv) does not perturb the radiation field; (v) nonhazardous to humans; and (vi) able to provide real‐time dosimetric information. Thermoluminescence dosimeter (TLD)^5^, ^6^, ^7^ is small in size, but requires a long series of pre‐ and postirradiation process. Radiochromic film^8^, ^9^, ^10^ has excellent dosimetric spatial resolution, is able to provide two‐dimensional (2D) dosimetric information^(11)^ and is easy to use, but it is not done in real‐time and may be affected by improper handling and scanner performance. Semiconductor detectors such as diode^12^, ^13^ and metal oxide semiconductor field effect transistor (MOSFET)^14^, ^15^, ^16^, ^17^, ^18^, ^19^ are able to achieve excellent spatial resolution with their small sensitive volumes. However, the energy, angle, temperature, and dose‐rate dependence of semiconductor detectors require rigorous characterization.

The dose deposited on a phantom or patient surface mainly comes from primary photon beam, backscattered radiation from the phantom, as well as radiation contamination from the accelerator. Radiation contamination arises from: (i) treatment head materials and (ii) treatment setup parameters such as source‐to‐surface distance (SSD), field size, and beam modifier to the surface dose.^(20)^ These contaminations will affect the dose in the buildup region. Therefore, it is essential to determine and know the effect of these treatment parameters.

Different terminologies, such as surface dose, skin dose, and entrance dose, have been used to describe the dose measured on the surface of a phantom or a human. The definitions for these terminologies differ according to the point of measurement on the patient or phantom. Surface dose is defined as the dose on the surface of the phantom or human, which is the interface between the air and the surface. Skin dose is defined as the dose at the depth of 0.07 mm.^(21)^ Entrance dose is defined as the dose given by the entrance beam at the depth of maximum dose.^(22)^

Characterization of a dosimeter is normally performed at a condition where charged particle equilibrium (CPE) condition exists.^12^, ^13^, ^19^ However, for *in vivo* skin dosimetry, the dosimeter should be characterized on the surface instead of the depth of maximum dose. This is because the dosimetric condition of skin surface and buildup region is different from the dosimetric condition at the depth of maximum dose. At the interface of two media (air and human tissue), CPE does not exist and there is a steep dose gradient in the buildup region. Therefore, characterization of a dosimeter on surface is needed prior to using it for *in vivo* skin dosimetry.

A MOSFET‐based dosimeter, the MO*Skin* detector was designed and prototyped by the Center for Medical Radiation Physics (CMRP) in the University of Wollongong (UoW). The advantages of the MO*Skin* detector, such as being small in size with submicron dosimetric volume which provides excellent dosimetry spatial resolution, as well as the ability to provide real‐time reading and instant readout, make it suitable for *in vivo* skin dosimetry measurement. The MO*Skin* detector has been characterized and been used for dose measurement in megavoltage radiotherapy and brachytherapy.^23^, ^24^, ^25^, ^26^, ^27^, ^28^, ^29^, ^30^

In this paper, a full characterization of the MO*Skin* detector on the surface of a phantom simulating the actual condition for *in vivo* skin dosimetry (where non‐CPE condition exist) was performed and reported. These include: (i) detector calibration, linearity, reproducibility; (ii) source to surface distance dependence; (iii) field size dependence; (iv) surface dose measurement; (v) angular dependence; and (vi) wedge response. Comparison and verifications were made with previous works with some extension, while benchmarking against different dosimeters that are available commercially and used extensively in radiotherapy centers.

## II. MATERIALS AND METHODS

### A. The MOS*kin* detector

**Figure 1:**
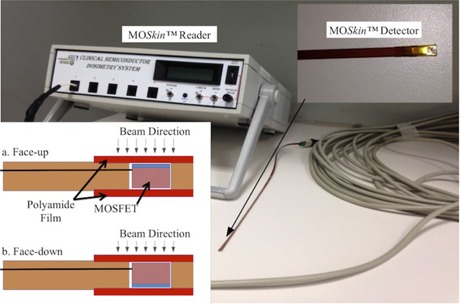
MO*Skin* system, MO*Skin* detector (top right), and the schematic diagram of MO*Skin* detector in (a) face‐up and (b) face‐down orientation.

The *MOSkin* system is shown in Fig. 1. The *MOSkin* detector is composed of hermetically sealed MOSFET dye with submicron thickness of the sensitive volume into Kapton pigtail strip with thickness of 0.55 mm using “drop‐in” packaging technology(^31^) (Fig. 1(a)). The thin reproducible polyamide film acts as an electrical connection and buildup for *MOSkin*, and gives a water‐equivalent depth (WED) of approximately 0.07 mm in tissue, making it a suitable dosimeter for skin dose measurement.^(24^) According to the International Commission on Radiological Protection (ICRP) publication,^(21^) the most radiosensitive layer of epidermis is located at tissue depth of approximately 0.07 mm. A detailed description of the *MOSkin* dosimetry system can be found in Kwan et al.^(24^) and Qi et al.^(26^) The readout process of *MOSkin* detector requires measurement of the voltage across the gate of the *MOSkin* detector under condition of the constant source‐drain current that is called the threshold voltage, $V_{\text{th}}$. The $V_{\text{th}}$ increases with accumulated radiation dose. The readout current corresponds to the thermostable point of the MOSFET to avoid errors associated with thermal instability of the $V_{\text{th}}$. The sensitivity of the *MOSkin* detector is defined as the shift of the $V_{\text{th}}$ with the absorption of 1 cGy of radiation dose (Eq. (1)). In this work, the *MOSkin* measurements were benchmarked against Markus ionization chamber (Markus type 23343 parallel plate ionization chamber; PTW, Freiburg, Germany) and/or GAFCHROMIC EBT2 film (International Specialty Products, Wayne, NJ). All measurements were carried out three times and the mean ± 1 SD of the readings were reported unless stated otherwise.

(1) $$Sensitivity=\frac{\Delta V_{th}}{1\;cGy}$$

where $\Delta V_{th}$ is the change of the threshold voltage in unit Volt (V).

### B. GAFCHROMIC EBT2 film preparation

GAFCHROMIC EBT2 films were cut into sizes of $1.5\;\times \;1.5\;{\text{cm}}^{2}$. They were scanned using a flatbed scanner (Epson 10000XL scanner; Epson America, Inc. Long Beach, CA) 24 hours after irradiation to allow for postirradiation color changes.^(32)^ The films were scanned in a reflection mode, at a resolution of 96 dots per inch (dpi), 48‐bits RGB format, and analyzed using ImageJ 1.46r software (National Institute of Health, Bethesda, MD). Care was taken to scan the films at the center of the scanner to avoid scanner‐induced nonuniformity. The films were also scanned in the same orientation to avoid film‐induced changes in pixel values.^(33^) Only the red channel was used for analysis. A region of interest (ROI) was selected at the center of the film. A set of standard films was irradiated to establish the calibration curve.

### C. Detector characterization

#### C.1 Calibration, linearity, and reproducibility

The *MOSkin* detectors were calibrated under a Varian Clinac 2100 C/D accelerator (Varian Medical System, Palo Alto, CA) using 6 MV photon beam under standard conditions (1.5 cm depth in $30\;\times \;30\;\times \;15\;{\text{cm}}^{3}$ solid water phantom, 100 cm source‐surface distance (SSD), and $10\;\times \;10\;{\text{cm}}^{2}$ field size). Sensitivities of the *MOSkin* detector have been determined. Linearity measurement of the *MOSkin* detector was determined for a dose range of 50 cGy to 300 cGy, with an increment of 50 cGy, and the reproducibility was assessed.

In this work, the buildup cap for the Markus ionization chamber was removed in order to position the chamber's effective measurement closer to the surface. Temperature and pressure correction factor, polarity effect correction factor, and ionization recombination correction factor were taken into account for Markus ionization chamber measurements. Parallel plate ionization chambers (Markus ionization chamber) are known to overrespond due to side scatter from the chamber's wall.^34^, ^35^, ^36^ In this work, Gerbi and Khan's correction(^36^) ((2), (3)) was only applied in the surface dose measurement (Material & Methods section C.4).

(2) $$P^{\prime }(d,E)=P(d,E)-\varepsilon (0,E)le^{-a(d/d_{\text{max}})}$$

(3) ε(0,E)=[−1.66+(1.982IR)][C−15.8]

where, $P^{\prime }(d,E)$ is the corrected PDD, P(d,E) is the measured PDD, *E* is the energy, *l* is the plate separation (2 mm for Markus PTW 23343), α is constant (5.5), *C* is the sidewall collector distance (0.35 mm for Markus PTW 23343), *IR* is the ionization ratio, and *d* is the depth of the chamber front window below the surface of phantom surface. The calculated ε(d,E) are 10.14 and 6.89 for 6 MV and 10 MV photon beams, respectively.

**Figure 2:**
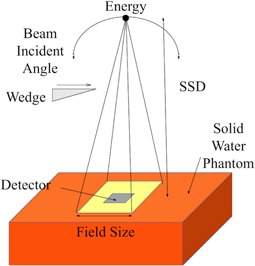
Schematic diagram of the standard setup of characterization of MO*Skin* detector on the surface of a solid water phantom.

Except for calibration and dose linearity measurement, all measurements were carried out on the surface of a solid water phantom. Characterization was carried out using a $30\;\times \;30\;\times \;15\;{\text{cm}}^{3}$ solid water phantom with a 6 MV photon beam, 100 cm SSD, and $10\;\times \;10\;{\text{cm}}^{2}$ field size (Fig. 2), unless stated otherwise. This setup is henceforth called the “standard surface setup”.

Cheung et al.^(37)^ has studied the temperature dependence of this MOSFET‐based detector. They reported that this detector shows a variation of 50 mV over the temperature range from 20°‐40°C. This variation is corresponding to about 10 cGy in dose. However, in order to get an accurate reading, the detector should be placed on phantom or patient approximately 60 s before measurement, to allow thermal equilibrium, and the reading are taken whilst the detector remains on the phantom or patient. The same precaution was also taken throughout this work to reduce the effect of temperature dependence of the detector.

#### C.2 Source‐to‐surface distance dependence

The MO*Skin* detector was positioned as per the “standard surface setup”. The response of the MO*Skin* detector for different distances from the source was measured with SSD, varying from 80 cm to 110 cm with 5 cm increments. One hundred MUs were delivered for each and repeated twice for all measured SSDs. Dose‐rate dependence of the MO*Skin* detector was also evaluated in this section. The dose rate at $d_{\text{max}}$ was calculated.

#### C.3 Field size dependence

The MO*Skin* detector was set up per “standard surface setup” and irradiated with different field sizes from $1\;\times \;1\;{\text{cm}}^{2}$ to $40\;\times \;40\;{\text{cm}}^{2}$ using 6 MV photon beam.

#### C.4 Surface dose measurement

The surface dose measured by MO*Skin* detector for 6 MV and 10 MV photon beams was evaluated. The measured dose by Markus ionization chamber was corrected, based on Gerbi and Khan's formulae.^(36)^

#### C.5 Angular dependence

Conventionally, angular dependence of the MOSFET was carried out at a depth where the CPE condition exists. Kwan et al.^(24)^ and Qi et al.^(29)^ have measured angular dependence of ± 2% for MO*Skin* detector in a cylindrical phantom, where the CPE exists, and Qi et al.^(26)^ have measured angular dependence of 3.1% on the surface of a solid water phantom. As reported in Scalchi et al.,^(17)^ full buildup setup where CPE exists gives better results than a surface setup.

Here we study the angular response of MO*Skin* detector placed on the surface of a phantom as the detector will be used for *in vivo* skin dose measurement where CPE does not exist. It is important to note that surface dose increases with beam incidence angle and correct measurements of the surface dose or skin dose for different angles of beam incidence are valuable for treatment planning system (TPS) verification. In particular, for the case of tangential beams, the angular response of the MO*Skin* detector will consist of the increased surface dose due to the beam incident angle and the intrinsic angular response of the MO*Skin*. The angular response of the MO*Skin* detector was assessed in face‐up and face‐down orientation, as shown in Figs. 1(a) and (b), by positioning the MO*Skin* detector per “standard surface setup”. One hundred MUs of 6 MV photon were delivered with the accelerator gantry rotated to the angles of 0° to 75°, with 15° increments.

#### C.6 Wedge response

In some clinical applications, such as conventional breast radiotherapy, beam modifier devices like physical wedge (PW) or dynamic wedge (DW)^38^, ^39^ may be required to tilt the dose profile, resulting in an angled isodose curve.^(38)^ Beam quality and dose rate of the incident photons may change due to the presence of the beam modifier device.^(40)^ For wedge response measurement, the MO*Skin* detector was irradiated using “standard surface setup”. One hundred MUs were delivered on the detector in an open field and subsequently with the application of PW and DW of 15°, 30°, 45°, and 60°.

## III. RESULTS & DISCUSSION

### A. Calibration, linearity and reproducibility

**Figure 3:**
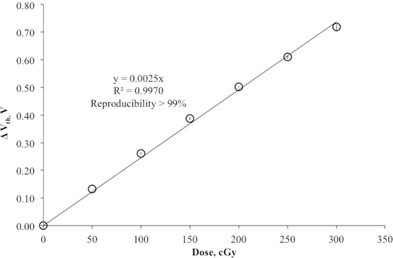
Linearity of MO*Skin* detector for the dose range of 0 cGy to 300 cGy.

**Figure 4:**
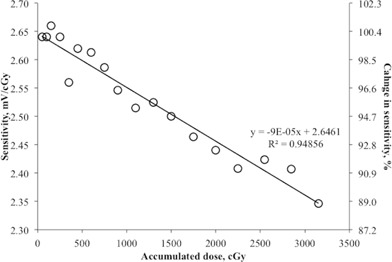
Change in sensitivity of MO*Skin* detector as a function of cumulative dose.

The MO*Skin* detector showed excellent reproducibility with deviation of less than 1% and excellent linearity $\left(R^{2}\;=\;0.997\right)$ for the dose range of 0 cGy to 300 cGy, as shown in Fig. 3. The reproducibility of the MO*Skin* detector was determined based on the average standard deviation (1 SD) of three repeated measurements of each dose level in linearity test. The dose linearity verification was carried out at this range because it was deemed to be within the range of a normal fractionated dose in radiotherapy. The average sensitivity of the MO*Skin* detectors in this study was 2.53 ± 0.03 mV/cGy for 6 MV photon beam. The sensitivity of the MO*Skin* detector as a function of cumulative dose was found to decrease by $9\;\times \;10^{‐2}\;\text{mV}/\text{cGy}$ for every 10 Gy of delivered dose (Fig. 4). The sensitivity of the MO*Skin* detectors is expected to decrease as the cumulative dose increases.^(41)^ Therefore, it is recommended that periodic recalibration be carried out throughout the detector's useful lifetime based on the accuracy needed. The readers are referred to Qi et al.^(23)^ for details of the MO*Skin* detector's lifetime.

### B. Source‐to‐surface distance dependence

**Figure 5:**
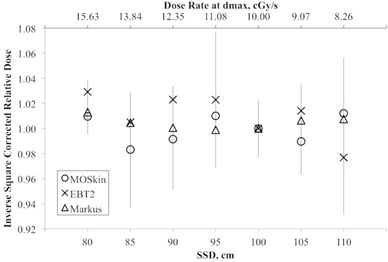
SSD and dose rate response of Markus ionization chamber, GAFCHROMIC EBT2 film and MO*Skin* detector corrected with ISCF and normalized to the response at 100 cm SSD. The error bar represents 1 SD of three sets of measurements.

Results of SSD dependence and dose rate dependence measurements for Markus ionization chamber, GAFCHROMIC EBT2 film, and the MO*Skin* detector are presented in Fig. 5. All measured doses were corrected with inverse square correction factor (ISCF) and normalized to the corrected dose at SSD 100 cm. An ideal SSD independent and dose rate independent dosimeter will have an “Inverse Square Corrected Relative Dose” equal to one.

From Fig. 5, the MO*Skin* detector, GAFCHROMIC EBT2 film, and Markus ionization chamber showed steady response over 80 cm to 110 cm SSD. The average variation of all SSDs for the MO*Skin* detector, GAFCHROMIC EBT2 film, and Markus ionization chamber was 0.1%, 1.0%, and 0.5%, respectively.

On the surface of the phantom, the dose deposited is not only due to the primary beam directed from the treatment head, but also from the contaminant electrons which are generated outside of the patient in the air and collimator. This may contribute to the SSD dependence of a skin dosimeter. This contamination is not sufficient to contribute to SSD dependence for large SSD as the electrons produced in the accelerator were of relatively high energy.^(20)^ Measurements at shorter SSD exposed the dosimeter to large amounts of low‐energy photons scattered by the components in the accelerator and would induce a slight over‐response of a dosimeter.

### C. Field size dependence

**Figure 6:**
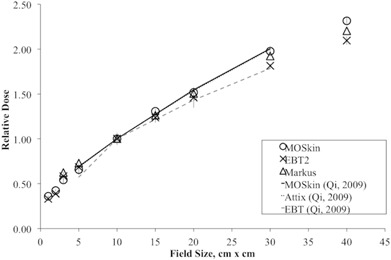
Field size response of the surface dose measured by MO*Skin* detector, GAFCHROMIC EBT2 film, and Markus ionization chamber normalized to response at $10\;\times \;10\;{\text{cm}}^{2}$ radiation field size. The average standard deviation of three sets of measurements for MO*Skin* detector, GAFCHROMIC EBT2 film, and Markus ionization chamber is 0.034, 0.052, and 0.001, respectively.

Qi et al.^(26)^ have also studied the field size dependence of the MO*Skin* detector on phantom surface for the field size of $5\;\times \;5\;{\text{cm}}^{2}$ to $30\;\times \;30\;{\text{cm}}^{2}$. In this study, the field sizes investigated were extended to include small field sizes of as small as $1\;\times \;1\;{\text{cm}}^{2}$ and larger field size of $40\;\times \;40\;{\text{cm}}^{2}$. The field size dependence of surface dose measured by MO*Skin* detector, as well as EBT2 film and Markus ionization chamber, is shown in Fig. 6. All readings were normalized to $10\;\times \;10\;{\text{cm}}^{2}$ field size. The three detectors showed an upward trend as field size increases. This is expected and is due to the increase in the backscattered radiation from the phantom and radiation contamination. The radiation contamination was mainly due to the scattered radiation from the flattening filter, with a small portion of scattered radiation as a function of field size.^(42)^ The result of MO*Skin* detector and Attix ionization chamber from Qi et al.^(26)^ are also presented in Fig. 6 for comparison. Surface dose increases measured with the MOSkin detectors rose from 0.36 to 2.32 times of the surface dose measurement at $10\;\times \;10\;{\text{cm}}^{2}$ for the field sizes from $1\;\times \;1\;{\text{cm}}^{2}$ to $40\;\times \;40\;{\text{cm}}^{2}$.

The dose deposited on the surface of the phantom is expected to be only 10%–20% of the maximum dose ($D_{\text{max}}$).^(38)^ As field size increases, the surface dose increases and, hence, there is a corresponding reduction in the surface dose gradient.^(17)^

All detectors measured increasing surface dose with increasing field size. For field size $<\;25\;\times \;25\;{\text{cm}}^{2}$, the MO*Skin* measurements are in good agreement with EBT2 film. MO*Skin* detector is advantageous for small field surface dosimetry compared to Markus ionization chamber due to the small sensitive volume of the detector. For field size $>\;25\;\times \;25\;{\text{cm}}^{2}$, MO*Skin* detector and Markus ionization chamber measurements showed an enhanced response, compared to the GAFCHROMIC EBT2 film. This may be due to the side scattering effect of Markus ionization chamber and energy dependence of the semiconductor (MO*Skin*) detector.

Good agreement between the MO*Skin* detector's response and Attix ionization chamber^(29)^ (average difference of 2%) has been observed due to close WED of the detectors used. Earlier reported results of field dependence measured with MO*Skin* detectors are in agreement (average difference of 3%) with presented results, confirming good reproducibility of WED of the MO*Skin* detectors.^(26)^

### D. Surface dose measurement

The average sensitivity of the MO*Skin* detectors was found to be 2.53 ± 0.03 mV/cGy and 2.50 ± 0.02 mV/cGy for 6 MV and 10 MV photon beams, respectively. The MO*Skin* detectors were 1.01% more sensitive in 6 MV photon beam compared to 10 MV photon beam.

Table 1 shows the measured surface dose of the MO*Skin* detector, GAFCHROMIC EBT2 film, and the Markus ionization chamber measurement for 6 MV and 10 MV photon beams. The surface doses for 6 MV photon measured by these detectors are higher than 10 MV photon due to the skin sparing effect of 10 MV photon.

Markus ionization chamber measured lowest surface doses for 6 MV and 10 MV photons, followed by the MO*Skin* detector and GAFCHROMIC EBT2 film. This can be explained by the WED of these detectors. Markus ionization chamber was assumed to have WED of 0 mm after the application of Gerbi and Khan's correction.^(36)^ MO *Skin* detector has a WED of 0.070 mm^(24)^ and GAFCHROMIC EBT2 film has a WED of 0.122 mm. The WED of GAFCHROMIC EBT2 film was determined based on the physical depth and density^43^, ^44^ from the surface to the center of the active layer.

In measuring the surface dose at the depth of 0 cm, the dose difference (%) between the MO*Skin* detector and Markus ionization chamber was found to be 4.44% and 1.74% for 6 MV and 10 MV photon beams, respectively (Table 1). It is expected because the dose gradient in build‐up region for 6 MV photon beam is steeper than that of 10 MV photon beam.

**Table 1:** Comparison of the surface dose (normalized to 100% the dose at $d_{\max}$) with Markus ionization chamber, GAFCHROMIC EBT2 film, and MO*Skin* detector for 6 MV and 10 MV photons

*Energy (MV)*	*Markus (%)*	*EBT2 (%)*	MO*Skin (%)*
6	15.83±0.03	23.01±0.07	20.27±0.03
10	11.82±0.00	17.89±0.04	13.55±0.04

### E. Angular dependence

**Figure 7:**
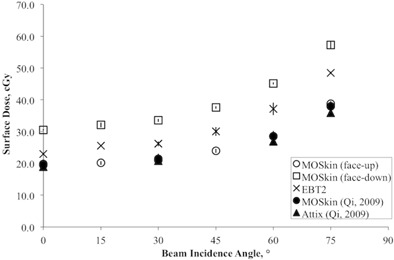
Relative surface dose measured with GAFCHROMIC EBT2 film and MO*Skin* detector as the function of beam incident angle and normalized to 1 at 0° beam incident angle with $10\;\times \;10\;{\text{cm}}^{2}$ field size. The error bar represents 1 SD of three sets of measurements. The average standard deviation of three sets of measurements for MO*Skin* detector and GAFCHROMIC EBT2 film is 0.033 and 0.036, respectively.

Figure 7 shows the results of the measured doses which have been normalized to the dose at 0° beam incident angle. The result of MO*Skin* detector and Attix ionization chamber from Qi et al.^(26)^ are also presented in Fig. 7 for comparison. As the beam incident angle increases, the measured surface dose increases because the region of charged particle equilibrium shifts toward the surface. This is in agreement with the results by Scalchi et al.^(17)^ and Qi et al.^(26)^

GAFCHROMIC EBT2 film, with its dosimetric properties of tissue equivalence and homogeneous material, is assumed to be of angular independence. Suchowerska et al.^(45)^ reported that GAFCHROMIC film shows intrinsic angular dependence of less than 1% when a measurement with the film surface is parallel and perpendicular to the beam direction. MO*Skin* detector in the face‐up orientation showed similar angular response trend to the GAFCHROMIC EBT2 film (Fig. 7). GAFCHROMIC EBT2 film measured higher dose as compared to MO*Skin* detector in face‐up orientation. This may be due to the difference of the WED these detectors (Results & Discussion section D).

When the MO*Skin* detector was used in the face‐down orientation for surface dose measurement, it showed a trend of overresponse when compared with MO*Skin* detector and GAFCHROMIC EBT2 film. Deviation between MO*Skin* detector in face‐up orientation and face‐down orientation is seen to be increasing as the beam incident angle increases. Maximum deviation of 18.5% was found at beam incident angle of 75°. The observed angular dependence between MO*Skin* detector in the face‐up orientation and face‐down orientation arises from the effect of the difference in the WED of the detector related to the asymmetric geometry of the detector (inherent anisotropy). For face‐down geometry, the WED is approximately 0.9 mm due to a 0.4 mm silicon substrate. Therefore, it is very important to identify the orientation of MO*Skin* detector when it is used for surface dose measurement. For WED 0.07 mm, MO*Skin* detector should be used in a face‐up mode. Present result was in good agreement with earlier reported result^(26)^ with maximum deviation of 2.2 % at angle 30°.

### F. Wedge response

**Figure 8:**
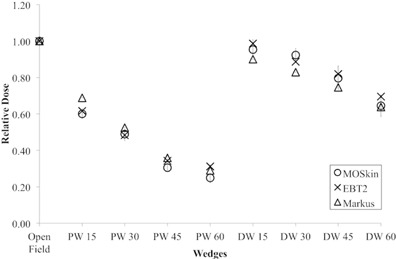
Wedges response measurement of Markus ionization chamber, GAFCHROMIC EBT2 film, and MO*Skin* detector and normalized to 1 at open field. The error bar represents 1 SD of three sets of measurements. The average standard deviation of three sets of measurements for MO*Skin* detector, GAFCHROMIC EBT2 film, and Markus ionization chamber is 0.033, 0.019, and 0.001, respectively.

The results of the wedge effect are summarized in Fig. 8. The doses were normalized to the dose measured with open field. The surface dose decreases as the wedge angle increases for both PW and DW. The average variation of MO*Skin* measurement with Markus ionization measurement for PW and DW was ‐5.5% and 5.1%, respectively. MO*Skin* measurement is in close agreement with GAFCHROMIC EBT2 film measurement. GAFCHROMIC EBT2 film responded higher for PW (2.8%) and DW (1.7%), respectively. This is in agreement with other scenarios of surface dose measurements due to higher WED of GAFCHROMIC EBT2 film in comparison with the MO*Skin* detector.

The presence of any materials between the radiation source and the phantom or patient will alter the dose to the build‐up region. In this work, constant MU (100 MUs) was given. The presence of physical wedge under the photon beam will harden the photon beam by absorbing scattered radiation.^(38)^ The presence of the wedge will also produce low energy scattered radiation.^(46)^

## IV. CONCLUSIONS

The surface dose measured with MO*Skin* detector was investigated on a phantom surface for different SSD, field sizes, surface dose, oblique beams, machine dose rates, and in the presence of the wedge in comparison with EBT2 film and Markus ionization chamber.

MO*Skin* detector showed deviation of less than 2% over a change of 80 – 110 cm SSD. For field size dependence, they are in agreement with Attix ionization chamber.^(29)^ Surface dose measured with the MO*Skin* detectors increases from 0.36 to 2.32 times of the surface dose measurement at $10\;\times \;10\;{\text{cm}}^{2}$ for the field sizes from $1\;\times \;1\;{\text{cm}}^{2}$ to $40\;\times \;40\;{\text{cm}}^{2}$. The dose difference between the MO*Skin* detector and Markus ionization chamber was 4.44% and 1.74% for 6 MV and 10 MV photon beams, respectively. This is due to the different WEDs for both detectors. The dose gradient in the buildup region of 6 MV photon beam is steeper than that of the 10 MV photon beam. When oblique beams are used, surface dose measured with the MO*Skin* detector increases up to 1.95 times of the normal beam incidence. For angular dependence, MO*Skin* detector in face‐up orientation is in agreement with Attix ionization chamber.^(26)^

Maximum deviation of 18.5% was found with face‐down orientation. The orientation (face‐up or face‐down) of the MO*Skin* detector must be taken into account when used for skin dose measurement. The MO*Skin* measurement is in close agreement with GAFCHROMIC EBT2 film for the measurement made under the presence of wedges.

MO*Skin* detector is suitable detector for *in vivo* skin dosimetry as compared to GAFCHROMIC EBT2 film because of its WED of 0.07 mm. However, due to the difference between the detector's WED and materials, neither can be used as true benchmarked for determining MO*Skin* detector accuracy as skin dosimeter. Monte Carlo calculation may provide a true benchmark tool for this comparison. However, this is not within the scope of this study.

## ACKNOWLEDGMENTS

We would like to acknowledge HIR Grant UM C/625/1/HIR/MOHE/CHAN/06 (H50001‐00‐A000020‐000001) and UMRG Grant RG507‐13HTM for supporting this research. We would also like to thank all the radiographers in the Clinical Oncology Unit of the University Malaya Medical Centre for their assistances in this study.

## Supporting information

Supplementary Material

Supplementary Material
