# Supplementary figures and images for: Characterization of MOSkin detector for in vivo skin dose measurement during megavoltage radiotherapy
Source: J Appl Clin Med Phys. 2014 Sep 8;15(5):120–32. doi: 10.1120/jacmp.v15i5.4869 (PMC5711095; doi:10.1120/jacmp.v15i5.4869)

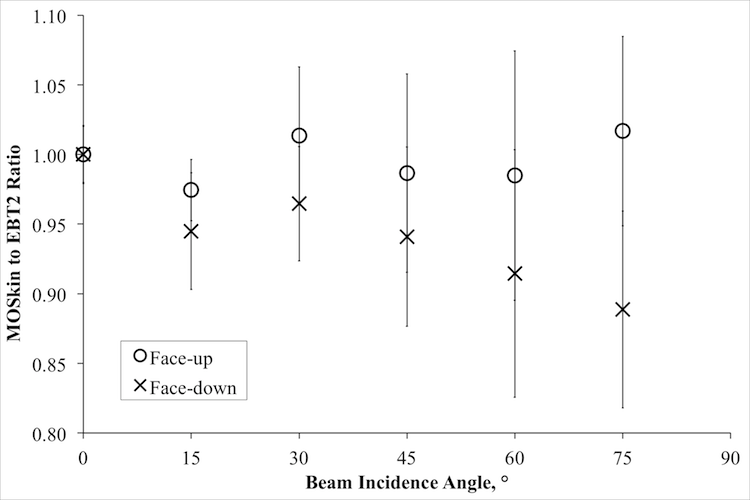

Supplement: Supplementary file 1 — Supplementary Material [file ACM2-15-120-s001.png]
